# Progress of Surgery

**Published:** 1874-10

**Authors:** Jno. E. Owens

**Affiliations:** Lecturer on Surgery, Rush Medical College, Chicago


ARTICLE II.—*Progress of Surgery.* By JNO. E. OWENS, M.D., Lecturer on Surgery, Rush Medical College, Chicago.

1. On the Nervous Mimicry of Diseases of Joints. By SIR JAMES PAGET, Bart., F. R. S., etc. (*Lancet*, November, 1873; *Braithwaite's Retrospect*, July, 1874.)

2. Nervous Mimicry of Diseases of the Spine. By SIR JAMES PAGET, Bart., F. R. S. (*Lancet*, Nov., 1873; *Braithwaite's Retrospect*, July, 1874.)

3. Injection of Chloral in Tetanus. By M. ORE, Professor of Bordeaux Medical School. (*Medical Times and Gazette; Braithwaite's Retrospect*, July, 1874.)

4. Fracture of the Clavicle Treated by Placing the Arm Behind the Back. By M. BROCA. (*Medical Press and Circular*, Dec. 24, 1873; *Braithwaite's Retrospect*, July, 1874.)

5. Amputation at the Hip-Joint by Esmarch's Method. By Dr. CHAS. I. GIBB, Consulting Surgeon to the Newcastle Infirmary. (*Lancet*, Jan. 31, 1874; *Braithwaite's Retrospect*, July, 1874.)

6. Extensive Disease of Knee-Joint; Amputation Through the Thigh; Bloodless Operation; Recovery. By J. KELLET SMITH, Esq., Stanley Hospital, Liverpool. (*Lancet*, Dec. 20, 1873; *Braithwaite's Retrospect*, July, 1874.)

7. On Bloodless Operations, as Illustrated by the Use of the Galvanic Cautery. By THOMAS BRYANT, Esq., Surgeon to Guy's Hospital. (*Lancet*, Feb., 1874; *Braithwaite's Retrospect*, July, 1874.)

8. Stricture of the Urethra. By FESSENDEN N. OTIS, M.D. (*New York Medical Journal*, April, 1874; *Philadelphia Medical Times*, May 16, 1874.)

9. Exploration of the Abdomen. By Prof. DENNING and others. (*Medical Press and Circular*, June 10, 1874.)

1. Mr. Paget, after reminding us of the fact that inflammation of a joint, either very acute or of long standing, can hardly exist without visible or tangible exudation in the joint-cavity, or in the surrounding textures; and after stating that swelling, frequently by the sides of ligamentum patella, is sometimes evident in a merely nervous joint—not considerable, but sufficient to make a mimicry of real disease much more close—a swelling similar to the puffiness that may come on in facial neuralgia, or the swelling and conjestion of the conjunctiva in some cases of orbital neuralgia, states, that, on the whole, the absence of swelling makes it very unlikely that a joint is really diseased. The presence of only a trivial swelling, when the nervous and muscular signs of disease are acute, or of long standing, is not indicative of joint inflammation. Such swelling is commonly transient and capricious, and the fallacy may be detected by observing, that at its greatest degree it is not, even after a long time or many repetitions, nearly proportionate to the pain or duration of the disease. A sensation of swelling is frequently complained of when no swelling really exists. A mere complaint of swelling will not deceive if the affected joint be compared with its fellow.

In this paper Sir James gives great prominence to temperature, as the sign most to be relied on in the diagnosis of real and nervous disease of joints. "It is so important to estimate it accurately, that I cannot too strongly urge you to be always studying it. You should feel with the broad surface of your hand every joint very watchfully; comparing each that is supposed to be diseased, with its fellow supposed or known to be healthy, you learn, as you certainly may, to detect even a small difference of temperature in even a small part of a joint."

A joint not above its natural temperature is not an inflamed joint; whatever may be the other signs of inflammation in it, it is not inflamed. Compare every suspected joint with its fellow. Compare the temperature of the joint with that of the rest of the limb, for the rest of the limb may be, through disease or long defective nutrition, cold; and if one joint in it be always not cold, though it may not be fairly called hot, this may arise from inflammation.

The certainty of diagnosis based on coldness is increased by a coincident dull purplish tint, which is commonly called blue or pink. Such colors may be present at joints long inflamed, but in these cases they are associated with over-heat.

2. If there be no considerable attendant illness, an intense and horrible pain in the spine does not point to serious disease of the spine. Such pain is not always merely " nervous " when it is the only wrong complained of. It may be due to aneurism or cancer. It is not a sign of spinal disease, unless when the pain of some slight disease is greatly exaggerated in a nervous constitution, or in the acutest form of inflammation of the vertebræ—a rare disease, and always associated with serious general illness and impaired mobility of the spine.

Excessive tenderness is characteristic of merely nervous disorder of the so-called spinal irritation, and is usually found at two or more parts of the spine. At these spots nervous patients cannot bear to be touched, and they writhe and flinch when the pressure is very gentle and quite superficial, and yet these tender spots are not painful in moving, coughing, or sneezing.

A merely nervous pain is usually variable, and is more dependent than the real disease on the disorders of distant organs. It is increased by fatigue, both bodily and mental. It is nearly a sure sign of nervous disorder, if pressure on the spine produces shivering or nausea, or a feeling of going-to-be-sick. It is a sign of nervous pain alone, if the pain has continued many weeks or months and nothing has come of it—no immobility of the spine or ribs, no paralysis, no fever, wasting or failure of the general health. When we find a patient carefully holding his head and neck, or any part of his back, very still, or turning or bending cautiously, we must look for disease of the spine. When the patient is able to tumble this way or that, to cough and to sneeze, without pain, we may be sure that organic spinal disease does not exist.

3. A man, after a wound of the finger, became the subject of tetanus, in consequence of which his mouth became so closed that no remedy could be administered. M. Ore threw an injection, containing ten grammes (154.30 grs.) into the veins. Peaceful sleep followed. This was followed by a second and third injection, with the effect of obtaining a sleep of eight hours.

4. M. Broca, adopting a suggestion made last year by Dr. Michel, recently treated a case of fracture of the clavicle, by placing the arm in a semi-flexed position behind the back, where it was retained by bandage for eighteen days, with the effect of completely adjusting the broken surfaces, and producing an excellent cure. The fracture was near the middle of the bone; was oblique, from above downwards, and from without inwards. A sling was subsequently used.

5. Dr. Gibb, having occasion to amputate the left leg of a youth, eighteen years old, at the hip-joint, at the suggestion of Dr. Page, the house-surgeon, employed Esmarch's method. The patient suffered from malignant disease of the femur. The elastic bandage was applied from the toes to the perineum, a good-sized pad of linen placed over the aorta, just below the umbilicus, and seven or eight turns of elastic tubing made over it. Not a drop of arterial blood was lost till the ligature was loosened, and then only one vessel spouted, the others having been secured. No inconvenience was experienced from the application of the tubing tightly around the belly.

6. The patient had suffered from knee-joint disease for four years. The history of the case shows that in spite of the excessive œdema which was said to exist, Esmarch's method most effectually succeeded in making a bloodless operation; and that, although the pressure was kept up for half an hour, there was no sloughing.

7. The disadvantages which Mr. Bryant associates with Esmarch's method are the following:

(*a.*) The prolonged and general capillary oozing of blood, from the tissues, following the removal of the elastic tubing.

(*b.*) The possibility that this secondary oozing may interfere with or prevent the immediate union of a wound.

(*c.*) The danger of injury to the larger nerves by undue compression. Langenbeck has recorded three instances in which, by this pressure applied to the upper extremity, some paralysis of the median nerve followed.

(*d.*) The practice does not commend itself in aged subjects, and in others in whom the vessels are diseased; for in such the complete obstruction to the flow of blood into the tissues may be followed by sloughing or want of action.

Mr. Bryant suggests that it would be wise to describe the operations performed by Esmarch's plan as examples of bloodless operating, reserving the term bloodless operations to those performed without blood from beginning to end.

With respect to the bloodless method by the elastic band, as suggested by Professor Dittel, one grave, practical objection is the fetor connected with the sloughing process; and unless its advocates can show that it possesses some equivalent advantages, it will never find a place in ordinary surgical practice. From 1872 to the end of 1873, Professor Dittel, by means of the elastic ligature, cured nævi, cases of anal fistule and sinuous passages. He has also removed by it a cancerous breast, amputated three legs, ligatured many arteries, and treated cases of hydrocele and phimosis, together with many examples of cancerous and other tumors. Sir Henry Thompson, its advocate in England, has applied it for the removal of a breast, which, by the way, did not come away for twenty-eight days. Mr. Bryant seems thoroughly convinced of the value of the galvanic cautery and its superiority over both Dittel and Esmarch's methods, for the performance of the bloodless operations. In comparison with this method (galvanic cautery) all other so-called bloodless methods are delusions. The battery must be in good working order, and the instruments complete and whole. The author of the paper has considered the use of the galvano-cautery under different headings, and demonstrated its value by a record of cases: operations on the tongue, penis and anus; the removal of tumors, the destruction of cancers, and the cure of lupus; the treatment of nævi.

In respect to operations on the tongue, since there are none in which, without its use, hemorrhage is more troublesome or dangerous, there are none with its use, which more satisfactorily illustrates its bloodless character. By the use of the galvano-ecraseur, carefully employed, no fear of bleeding need disturb the mind of the operator; and what was formerly a very serious measure has become a comparatively simple one.

By means of Mr. Bryant's method four cases of lupus are reported cured, viz.: one of four years' standing, cured in twenty-four days; one of four years' standing, cured in twenty-seven days; one of twenty years' standing, cured in twenty days; the fourth was also successfully treated. In conclusion, it is worthy to be mentioned that seven cases of epithelial cancer, and twelve of nævi are reported cured by the same method.

8. Dr. Otis makes the assertion that stricture of the urethra is absolutely within the reach of curative measures; that, if completely divided, and this division maintained until the healing process has been completed, no contraction can ever take place. He does not deem the division of the stricture more hazardous than rapid, permanent or temporary dilatation.

9. The kind of examination referred to consists in the introduction of the hand, well oiled, into the rectum, by accomplishing the progressive dilatation of the anus, by means of the gradual introduction of two, three, and, lastly, four fingers into the rectum as far as the iliac flexure, and even into the colon, so as to explore the pelvis, and even the whole abdomen as far as the diaphragm. Usually there follows some incontinence of fæces during a few days following the operation. Such exploration is undertaken in cases of obstruction of the bowels, to ascertain the exact seat of the obstruction. The danger of perforating a diseased intestine in such explorations, must not be overlooked.

In the discussion which followed the mention of this operation by Professor Denning before the New York Academy, Dr. C. Leale stated that he had practiced the operation with the intention of exciting the solar plexus in a patient who had attempted suicide with chloroform, and was apparently dead. The operator gradually attained the pillars of the diaphragm, and, after having mechanically excited the plexus, had the satisfaction of seeing the respirations become more vigorous and the patient restored to life. The dilatation of the anus amounted to six inches, and the hand arrived at four inches above the umbilicus—sixteen inches above the anus.

Dr. Morton performed the same operation on a patient aged twenty-six years, in whom he suspected the presence of a stone in the ureter.

Professor Peaslee admitted its utility in explorations of the pelvic organs, but thought that pushing the hand further might expose the patient to danger.

